# The genome sequence of the chalcid wasp, *Chalcis sispes *Linnaeus, 1761

**DOI:** 10.12688/wellcomeopenres.22693.1

**Published:** 2024-07-22

**Authors:** Olga Sivell, Ryan Mitchell, Judy Webb, Gavin R. Broad, Natural History Museum Genome Acquisition Lab, Darwin Tree of Life Barcoding collective, Wellcome Sanger Institute Tree of Life Management, Samples and Laboratory team, Wellcome Sanger Institute Scientific Operations: Sequencing Operations, Wellcome Sanger Institute Tree of Life Core Informatics team, Tree of Life Core Informatics collective, Darwin Tree of Life Consortium

**Affiliations:** 1 Natural History Museum, London, England, UK; 2 Independent researcher, Sligo, Sligo County, Ireland

**Keywords:** Chalcis sispes, chalcid wasp, genome sequence, chromosomal, Hymenoptera

## Abstract

We present a genome assembly from an individual female *Chalcis sispes* (chalcid wasp; Arthropoda; Insecta; Hymenoptera; Chalcididae). The genome sequence is 412.4 megabases in span. Most of the assembly is scaffolded into 6 chromosomal pseudomolecules. The mitochondrial genome has also been assembled and is 15.9 kilobases in length.

## Species taxonomy

Eukaryota; Opisthokonta; Metazoa; Eumetazoa; Bilateria; Protostomia; Ecdysozoa; Panarthropoda; Arthropoda; Mandibulata; Pancrustacea; Hexapoda; Insecta; Dicondylia; Pterygota; Neoptera; Endopterygota; Hymenoptera; Apocrita; Proctotrupomorpha; Chalcidoidea; Chalcididae; Chalcidinae; *Chalcis*; *Chalcis sispes* Linnaeus, 1761 (NCBI:txid1118640).

## Background

*Chalcis sispes* is an extraordinary-looking parasitoid wasp. The family Chalcididae are instantly recognisable, at least in the British fauna, by their hugely expanded hind femora with ventral teeth and their curved hind tibiae. In the north temperate zone there are few species of Chalcididae and in Britain only ten species are known ( Dale-Skey *et al.*, 2016). Ferrière & Kerrich (1958) provide a key to the British species which is mostly still relevant. The metasoma of *Chalcis* has a long petiole, and the hind coxa is also very long. *Chalcis sispes* is the most widespread of the three British species and like other *Chalcis* species inhabits the edges of water where its host stratiomyid flies can be found. Eggs of *Stratiomys* flies are laid in masses on emergent vegetation and *C. sispes* oviposits into the eggs, completing development in the larva when it emerges from the water to pupate ( Burks, 1979).

The family Chalcididae is morphologically and biologically diverse, especially in the tropics, and the huge hind legs, which have also evolved in a variety of other chalcidoids, probably fulfil a variety of functions. At least in *Chalcis*, the legs are used as robust props while manipulating the egg hosts with its fore and mid legs. They are also used in fighting, as females sometimes defend host egg masses against other females ( Cowan, 1979). The eggs of *C. sispes* are rather distinctive, with stalks at either pole ( Henneguy, 1891).

This first complete genome for the family Chalcididae will help unravel the evolutionary innovations which have enabled the extraordinary diversification of the Chalcidoidea ( Cruaud *et al.*, 2024).

## Genome sequence report

**Figure 1.**
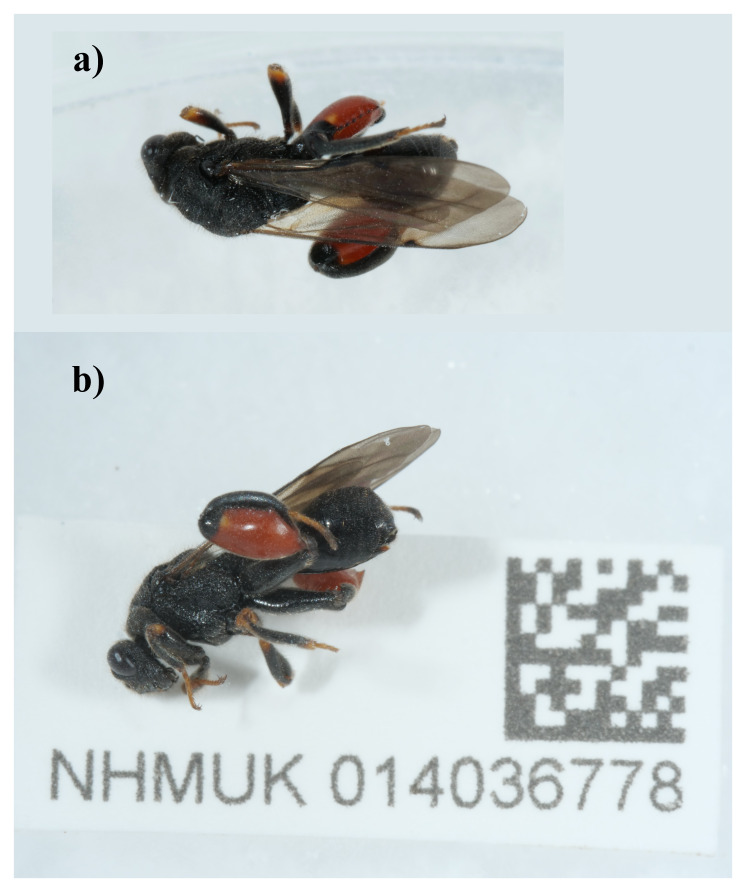
Photographs of the *Chalcis sispes* specimen (NHMUK014036778, iyChaSisp2) used for genome sequencing.

The genome was sequenced from a female *Chalcis sispes* ( Figure 1) collected from Parsonage Moor, Abingdon, England (51.69, –1.33). A total of 57-fold coverage in Pacific Biosciences single-molecule HiFi long reads was generated. Primary assembly contigs were scaffolded with chromosome conformation Hi-C data. Manual assembly curation corrected 53 missing joins or mis-joins and removed 6 haplotypic duplications, reducing the scaffold number by 61.11%, and increasing the scaffold N50 by 4.70%.

**Figure 2.**
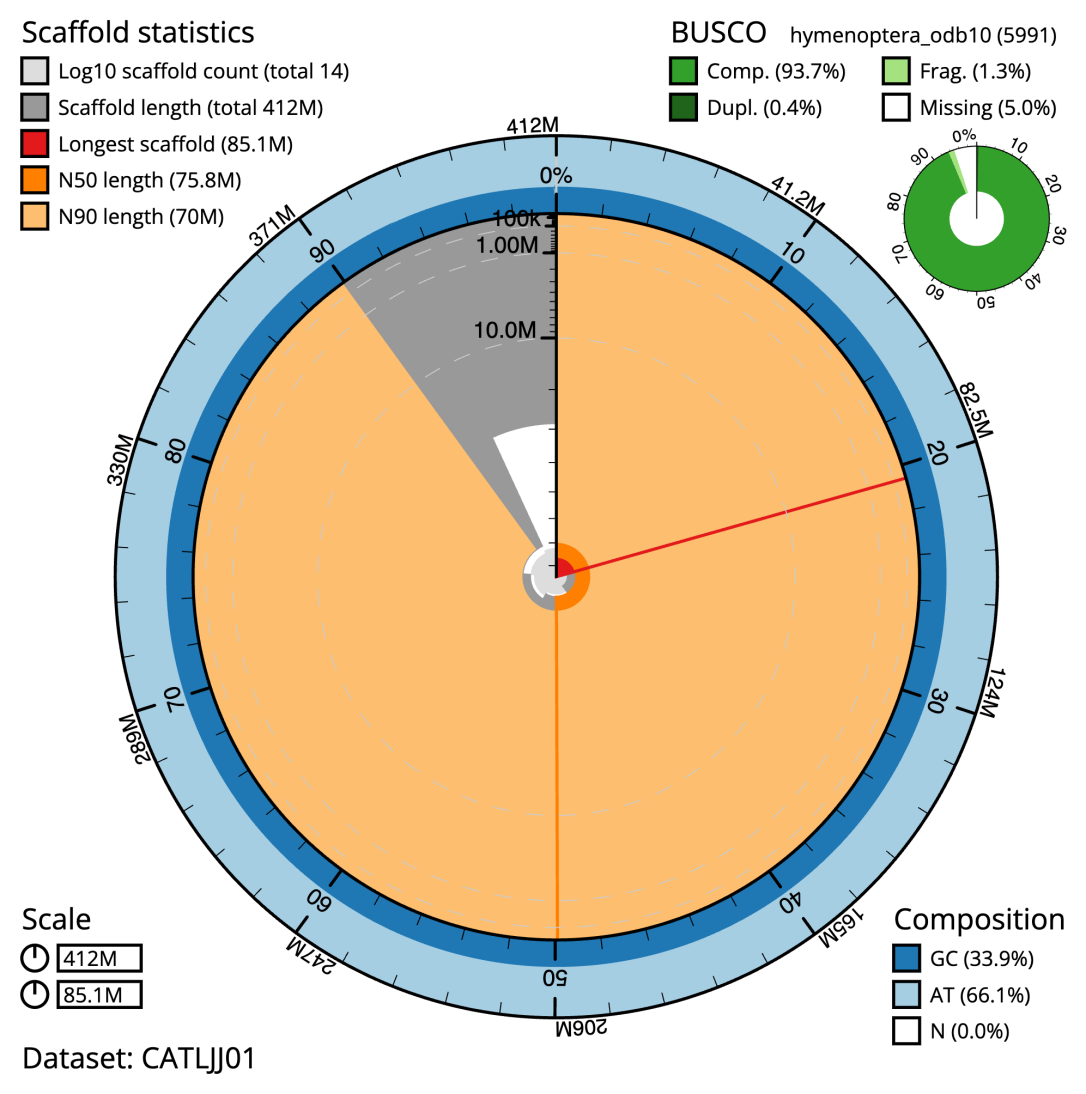
Genome assembly of *Chalcis sispes*, iyChaSisp2.1: metrics. The BlobToolKit Snailplot shows N50 metrics and BUSCO gene completeness. The main plot is divided into 1,000 size-ordered bins around the circumference with each bin representing 0.1% of the 412,410,240 bp assembly. The distribution of scaffold lengths is shown in dark grey with the plot radius scaled to the longest scaffold present in the assembly (85,135,257 bp, shown in red). Orange and pale-orange arcs show the N50 and N90 scaffold lengths (75,805,882 and 69,969,611 bp), respectively. The pale grey spiral shows the cumulative scaffold count on a log scale with white scale lines showing successive orders of magnitude. The blue and pale-blue area around the outside of the plot shows the distribution of GC, AT and N percentages in the same bins as the inner plot. A summary of complete, fragmented, duplicated and missing BUSCO genes in the hymenoptera_odb10 set is shown in the top right. An interactive version of this figure is available at https://blobtoolkit.genomehubs.org/view/CATLJJ01/dataset/CATLJJ01/snail.

**Figure 3.**
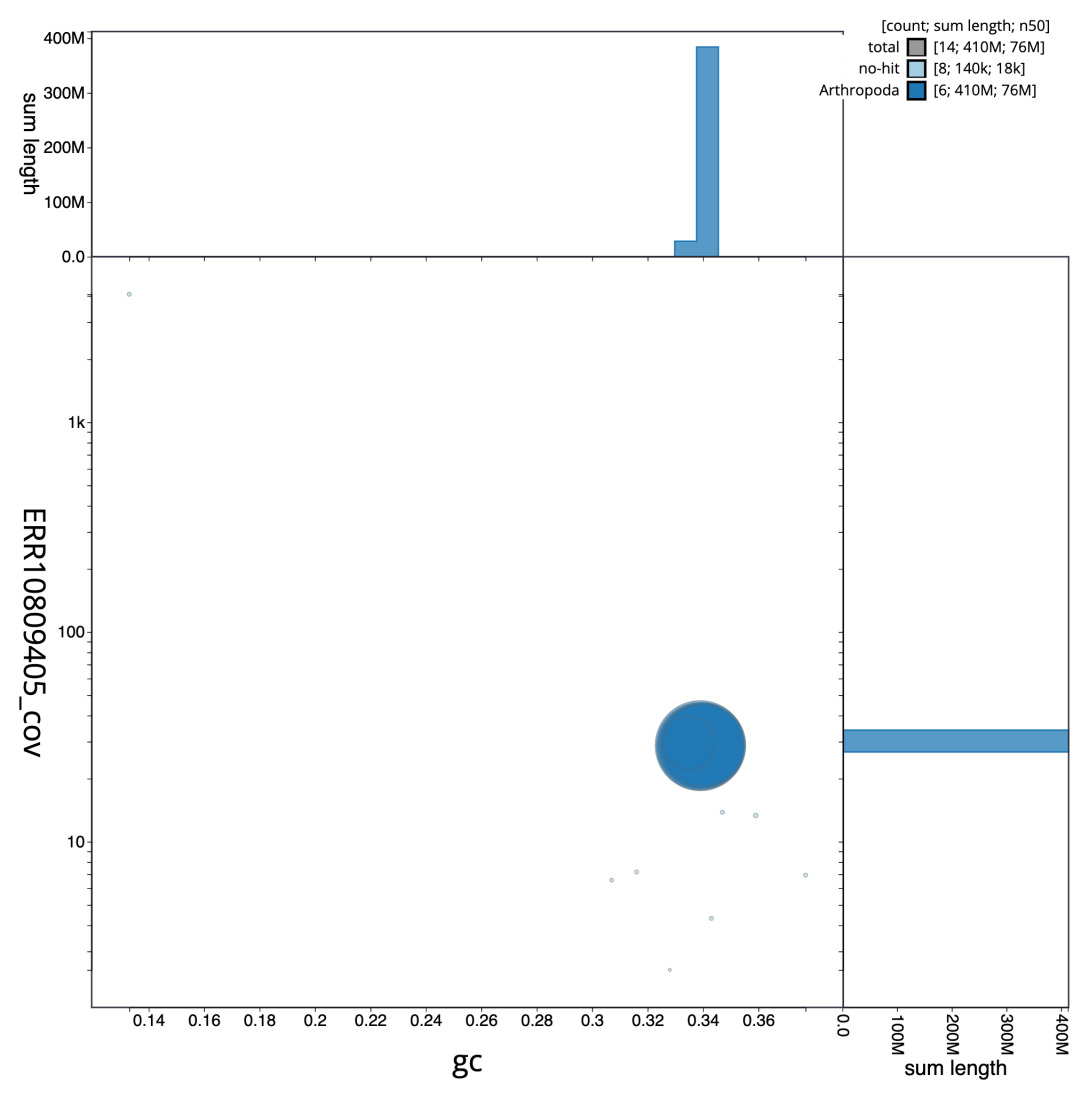
Genome assembly of *Chalcis sispes*, iyChaSisp2.1: BlobToolKit GC-coverage plot. Scaffolds are coloured by phylum. Circles are sized in proportion to scaffold length. Histograms show the distribution of scaffold length sum along each axis. An interactive version of this figure is available at https://blobtoolkit.genomehubs.org/view/CATLJJ01/dataset/CATLJJ01/blob.

**Figure 4.**
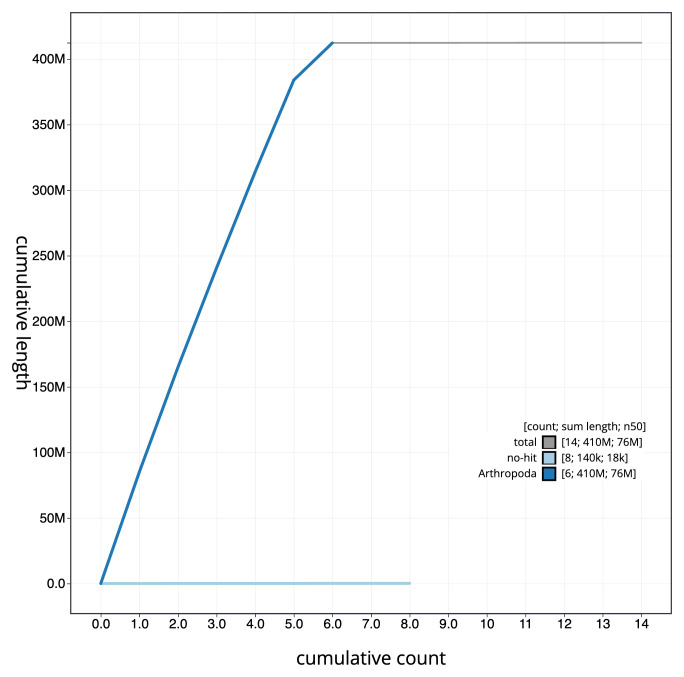
Genome assembly of *Chalcis sispes*, iyChaSisp2.1: BlobToolKit cumulative sequence plot. The grey line shows cumulative length for all scaffolds. Coloured lines show cumulative lengths of scaffolds assigned to each phylum using the buscogenes taxrule. An interactive version of this figure is available at https://blobtoolkit.genomehubs.org/view/CATLJJ01/dataset/CATLJJ01/cumulative.

**Figure 5.**
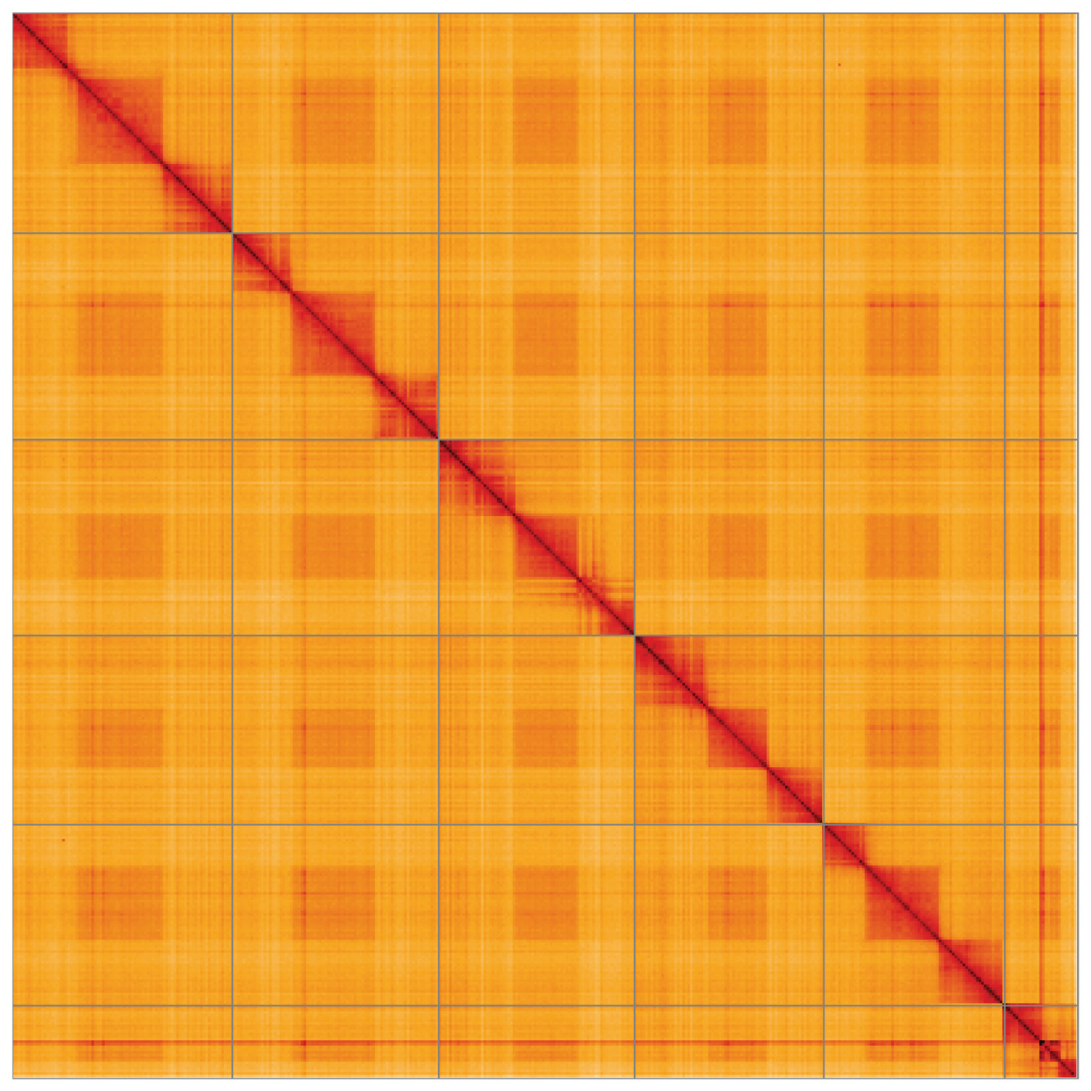
Genome assembly of *Chalcis sispes*, iyChaSisp2.1: Hi-C contact map of the iyChaSisp2.1 assembly, visualised using HiGlass. Chromosomes are shown in order of size from left to right and top to bottom. An interactive version of this figure may be viewed at https://genome-note-higlass.tol.sanger.ac.uk/l/?d=dbWjnZj_RYOT9mV81R5qaQ.

The final assembly has a total length of 412.4 Mb in 13 sequence scaffolds with a scaffold N50 of 75.8 Mb ( Table 1). The snail plot in Figure 2 provides a summary of the assembly statistics, while the distribution of assembly scaffolds on GC proportion and coverage is shown in Figure 3. The cumulative assembly plot in Figure 4 shows curves for subsets of scaffolds assigned to different phyla. Most (99.96%) of the assembly sequence was assigned to 6 chromosomal-level scaffolds. Chromosome-scale scaffolds confirmed by the Hi-C data are named in order of size ( Figure 5; Table 2). While not fully phased, the assembly deposited is of one haplotype. Contigs corresponding to the second haplotype have also been deposited. The mitochondrial genome was also assembled and can be found as a contig within the multifasta file of the genome submission.

**Table 1.** Genome data for *Chalcis sispes*, iyChaSisp2.1.

Project accession data			
Assembly identifier	iyChaSisp2.1		
Species	*Chalcis sispes*		
Specimen	iyChaSisp2		
NCBI taxonomy ID	1118640		
BioProject	PRJEB59203		
BioSample ID	SAMEA11025062		
Specimen information			
**Technology**	**ToLID**	**BioSample ** **accession**	**Organism part**
**PacBio long read sequencing**	iyChaSisp2	SAMEA11025310	Whole organism
**Hi-C sequencing**	iyChaSisp1	SAMEA11025285	Whole organism
Sequencing information			
**Platform**	**Run accession**	**Read count**	**Base count (Gb)**
**Hi-C Illumina NovaSeq 6000**	ERR10818308	7.06e+08	106.56
**PacBio Sequel IIe**	ERR10809405	2.21e+06	24.15
Assembly metrics *			*Benchmark*
Consensus quality (QV)	59.9		*≥ 50*
*k*-mer completeness	100.0%		*≥ 95%*
BUSCO **	C:93.7%[S:93.2%,D:0.4%], F:1.3%,M:5.0%,n:5,991		*C ≥ 95%*
Percentage of assembly mapped to chromosomes	99.96%		*≥ 95%*
Sex chromosomes	None		*localised homologous pairs*
Organelles	Mitochondrial genome: 15.9 kb		*complete single alleles*
Genome assembly			
Assembly accession	GCA_949987625.1		
*Accession of alternate haplotype*	GCA_950005085.1		
Span (Mb)	412.4		
Number of contigs	457		
Contig N50 length (Mb)	2.0		
Number of scaffolds	13		
Scaffold N50 length (Mb)	75.8		
Longest scaffold (Mb)	85.14		

* Assembly metric benchmarks are adapted from column VGP-2020 of “Table 1: Proposed standards and metrics for defining genome assembly quality” from ( Rhie *et al.*, 2021).

** BUSCO scores based on the hymenoptera_odb10 BUSCO set using version 5.3.2. C = complete [S = single copy, D = duplicated], F = fragmented, M = missing, n = number of orthologues in comparison. A full set of BUSCO scores is available at https://blobtoolkit.genomehubs.org/view/CATLJJ01/dataset/CATLJJ01/busco.

**Table 2.** Chromosomal pseudomolecules in the genome assembly of *Chalcis sispes*, iyChaSisp2.

INSDC accession	Chromosome	Length (Mb)	GC%
OX465094.1	1	85.14	34.0
OX465095.1	2	79.82	34.0
OX465096.1	3	75.81	34.0
OX465097.1	4	73.21	34.0
OX465098.1	5	69.97	34.0
OX465099.1	6	28.33	33.5
OX465100.1	MT	0.02	13.5

The estimated Quality Value (QV) of the final assembly is 59.9 with *k*-mer completeness of 100.0%, and the assembly has a BUSCO v5.3.2 completeness of 93.7% (single = 93.2%, duplicated = 0.4%), using the hymenoptera_odb10 reference set ( *n* = 5,991).

Metadata for specimens, barcode results, spectra estimates, sequencing runs, contaminants and pre-curation assembly statistics are given at https://links.tol.sanger.ac.uk/species/1118640.

## Methods

### Sample acquisition and nucleic acid extraction

The *Chalcis sispes* specimens used for genome sequencing (specimen ID NHMUK014036778, ToLID iyChaSisp2) and Hi-C sequencing (specimen ID NHMUK014036779, ToLID iyChaSisp1) were collected from Parsonage Moor, Abingdon, England, UK (latitude 51.69, longitude –1.33) on 2021-06-19 using an aerial net. The specimens were collected by Olga Sivell (Natural History Museum) and Ryan Mitchell (independent researcher) and identified by Ryan Mitchell and Judy Webb (Natural History Museum), and then preserved in liquid nitrogen.

The workflow for high molecular weight (HMW) DNA extraction at the WSI includes a sequence of core procedures: sample preparation; sample homogenisation, DNA extraction, fragmentation, and clean-up. In sample preparation, the iyChaSisp2 sample was weighed and dissected on dry ice ( Jay *et al.*, 2023). Whole organism tissue was homogenised using a PowerMasher II tissue disruptor ( Denton *et al.*, 2023a). 

HMW DNA was extracted in the WSI Scientific Operations core using the Automated MagAttract v2 protocol ( Oatley *et al.*, 2023). HMW DNA was sheared into an average fragment size of 12–20 kb in a Megaruptor 3 system with speed setting 31 ( Bates *et al.*, 2023). Sheared DNA was purified by solid-phase reversible immobilisation ( Strickland *et al.*, 2023): in brief, the method employs a 1.8X ratio of AMPure PB beads to sample to eliminate shorter fragments and concentrate the DNA. The concentration of the sheared and purified DNA was assessed using a Nanodrop spectrophotometer and Qubit Fluorometer and Qubit dsDNA High Sensitivity Assay kit. Fragment size distribution was evaluated by running the sample on the FemtoPulse system.

Protocols developed by the Wellcome Sanger Institute (WSI) Tree of Life core laboratory have been deposited on protocols.io ( Denton *et al.*, 2023b).

### Sequencing

Pacific Biosciences HiFi circular consensus DNA sequencing libraries were constructed according to the manufacturers’ instructions. DNA sequencing was performed by the Scientific Operations core at the WSI on a Pacific Biosciences SEQUEL II instrument. Hi-C data were also generated from whole organism tissue of iyChaSisp1 using the Arima v2 kit. The Hi-C sequencing was performed using paired-end sequencing with a read length of 150 bp on the Illumina NovaSeq 6000 instrument.

### Genome assembly, curation and evaluation

Assembly was carried out with Hifiasm ( Cheng *et al.*, 2021) and haplotypic duplication was identified and removed with purge_dups ( Guan *et al.*, 2020). The assembly was then scaffolded with Hi-C data ( Rao *et al.*, 2014) using YaHS ( Zhou *et al.*, 2023). The assembly was checked for contamination and corrected using the gEVAL system ( Chow *et al.*, 2016) as described previously ( Howe *et al.*, 2021). Manual curation was performed using gEVAL, HiGlass ( Kerpedjiev *et al.*, 2018) and PretextView ( Harry, 2022). The mitochondrial genome was assembled using MitoHiFi ( Uliano-Silva *et al.*, 2023), which runs MitoFinder ( Allio *et al.*, 2020) or MITOS ( Bernt *et al.*, 2013) and uses these annotations to select the final mitochondrial contig and to ensure the general quality of the sequence.

A Hi-C map for the final assembly was produced using bwa-mem2 ( Vasimuddin *et al.*, 2019) in the Cooler file format ( Abdennur & Mirny, 2020). To assess the assembly metrics, the *k*-mer completeness and QV consensus quality values were calculated in Merqury ( Rhie *et al.*, 2020). This work was done using Nextflow ( Di Tommaso *et al.*, 2017) DSL2 pipelines “sanger-tol/readmapping” ( Surana *et al.*, 2023a) and “sanger-tol/genomenote” ( Surana *et al.*, 2023b). The genome was analysed within the BlobToolKit environment ( Challis *et al.*, 2020) and BUSCO scores ( Manni *et al.*, 2021; Simão *et al.*, 2015) were calculated.

Table 3 contains a list of relevant software tool versions and sources.

**Table 3.** Software tools: versions and sources.

Software tool	Version	Source
BlobToolKit	4.1.7	https://github.com/blobtoolkit/blobtoolkit
BUSCO	5.3.2	https://gitlab.com/ezlab/busco
gEVAL	N/A	https://geval.org.uk/
Hifiasm	0.16.1-r375	https://github.com/chhylp123/hifiasm
HiGlass	1.11.6	https://github.com/higlass/higlass
Merqury	MerquryFK	https://github.com/thegenemyers/MERQURY.FK
MitoHiFi	2	https://github.com/marcelauliano/MitoHiFi
PretextView	0.2	https://github.com/sanger-tol/PretextView
purge_dups	1.2.3	https://github.com/dfguan/purge_dups
sanger-tol/genomenote	v1.0	https://github.com/sanger-tol/genomenote
sanger-tol/readmapping	1.1.0	https://github.com/sanger-tol/readmapping/tree/1.1.0
YaHS	1.1a.2	https://github.com/c-zhou/yahs

### Wellcome Sanger Institute – Legal and Governance

The materials that have contributed to this genome note have been supplied by a Darwin Tree of Life Partner. The submission of materials by a Darwin Tree of Life Partner is subject to the **‘Darwin Tree of Life Project Sampling Code of Practice’**, which can be found in full on the Darwin Tree of Life website here. By agreeing with and signing up to the Sampling Code of Practice, the Darwin Tree of Life Partner agrees they will meet the legal and ethical requirements and standards set out within this document in respect of all samples acquired for, and supplied to, the Darwin Tree of Life Project. 

Further, the Wellcome Sanger Institute employs a process whereby due diligence is carried out proportionate to the nature of the materials themselves, and the circumstances under which they have been/are to be collected and provided for use. The purpose of this is to address and mitigate any potential legal and/or ethical implications of receipt and use of the materials as part of the research project, and to ensure that in doing so we align with best practice wherever possible. The overarching areas of consideration are:

●   Ethical review of provenance and sourcing of the material

●   Legality of collection, transfer and use (national and international) 

Each transfer of samples is further undertaken according to a Research Collaboration Agreement or Material Transfer Agreement entered into by the Darwin Tree of Life Partner, Genome Research Limited (operating as the Wellcome Sanger Institute), and in some circumstances other Darwin Tree of Life collaborators.

## Data Availability

European Nucleotide Archive: *Chalcis sispes*. Accession number PRJEB59203; https://identifiers.org/ena.embl/PRJEB59203 ( Wellcome Sanger Institute, 2023). The genome sequence is released openly for reuse. The *Chalcis sispes* genome sequencing initiative is part of the Darwin Tree of Life (DToL) project. All raw sequence data and the assembly have been deposited in INSDC databases. The genome will be annotated using available RNA-Seq data and presented through the Ensembl pipeline at the European Bioinformatics Institute. Raw data and assembly accession identifiers are reported in Table 1.

## Author information

Members of the Natural History Museum Genome Acquisition Lab are listed here: https://doi.org/10.5281/zenodo.7139035.

Members of the Darwin Tree of Life Barcoding collective are listed here: https://doi.org/10.5281/zenodo.4893703.

Members of the Wellcome Sanger Institute Tree of Life Management, Samples and Laboratory team are listed here: https://doi.org/10.5281/zenodo.10066175.

Members of Wellcome Sanger Institute Scientific Operations: Sequencing Operations are listed here: https://doi.org/10.5281/zenodo.10043364.

Members of the Wellcome Sanger Institute Tree of Life Core Informatics team are listed here: https://doi.org/10.5281/zenodo.10066637.

Members of the Tree of Life Core Informatics collective are listed here: https://doi.org/10.5281/zenodo.5013541.

Members of the Darwin Tree of Life Consortium are listed here: https://doi.org/10.5281/zenodo.4783558.
