# Supplementary material for: The genome sequence of the chalcid wasp, Chalcis sispes Linnaeus, 1761
Source: Wellcome Open Res. 2024 Jul 22;9:391. [Version 1] doi: 10.12688/wellcomeopenres.22693.1 (PMC11443186; doi:10.12688/wellcomeopenres.22693.1)
Supplement: Peer review history

# Reviewer report: Reviewer response for version 1

**DOI:** 10.21956/wellcomeopenres.24994.r100221

**Published:** 2024-09-30

**Authors:** Qingsong Zhou

**Affiliations:** 1 Chinese Academy of Sciences, Beijing, China

Olga et al. present a genome assembly of *Chalcis sispes *utilizing Pacific HIFI long reads. The background of the species *Chalcis sispes * is thoroughly described. The small size of parasitoid wasps poses challenges in extracting sufficient DNA for genome assembly; however, the DNA extraction method employed in this study will serve as a valuable reference for genomic research on other parasitoid wasps. Overall, the genome assembly demonstrates high quality, with a genome size of 412.4 MB and a scaffold N50 of 75.8 Mb. This genome assembly represents the first for the family Chalcididae and will enhance the genomic data available for understanding the evolutionary history and patterns of Chalcidoidea species.

 Although Hifiasm does not require polishing tools, the BUSCO score of this genome assembly indicates that an additional polishing step using genome survey data may be necessary to further improve the assembly quality.

 Besides, the authors stated that the genome scaffolds were assigned to six chromosomal pseudomolecules using Hi-C data; however, it is hard to identify these six chromosomes according to the Hi-C contact map. It is essential to verify the number of chromosomes through multiple Hi-C scaffolding methods.

Are sufficient details of methods and materials provided to allow replication by others?

Yes

Is the rationale for creating the dataset(s) clearly described?

Yes

Are the datasets clearly presented in a useable and accessible format?

Yes

Are the protocols appropriate and is the work technically sound?

Yes

Reviewer Expertise:

entomology, genomics

I confirm that I have read this submission and believe that I have an appropriate level of expertise to confirm that it is of an acceptable scientific standard.

# Reviewer report: Reviewer response for version 1

**DOI:** 10.21956/wellcomeopenres.24994.r95316

**Published:** 2024-09-20

**Authors:** Legan Andrew Wesley

**Affiliations:** 1 Entomology, The University of Arizona, Tucson, Arizona, USA

This Data Note reports a 412.4 Mb genome assembly of a single individual female chalcidid wasp, *Chalcis sispes*, from South England, assembled from HiFi and Hi-C data.

 It was a good choice of taxon because only one other member of Chalcididae has had genome sequenced to my knowledge: *Brachymeria lasus* by workers at Anhui Normal University using nanopore sequencing (GenBank accession GCA_040285435.1).

 Gene annotations were not reported here but I assume annotation is in-progress since RNAseq data is in the process of being added to the Tree of Life QC page for this species (https://links.tol.sanger.ac.uk/species/1118640). ENA Project: PRJEB59202, Experiment: ERX12864170 contains RNA-Seq data for *Chalcis sispes*, which could inform gene model prediction. It would be interesting to know the BUSCO scores for the *Chalcis sispes* predicted proteins because the BUSCO estimated completeness (C) of the assembly reported here was 93.7% - slightly below the 95% C benchmark.

 The genome is highly contiguous (chromosome-level), and with 57x coverage I would expect the HiFi reads to provide an accurate estimate of the genome sequence. Since genome completeness and accuracy do not seem to be issues, I wonder what might account for the slightly lower than ideal BUSCO completeness estimate.

Are sufficient details of methods and materials provided to allow replication by others?

Yes

Is the rationale for creating the dataset(s) clearly described?

Yes

Are the datasets clearly presented in a useable and accessible format?

Yes

Are the protocols appropriate and is the work technically sound?

Yes

Reviewer Expertise:

comparative genomics, animal behavior, chemical ecology, multi-gene family evolution

I confirm that I have read this submission and believe that I have an appropriate level of expertise to confirm that it is of an acceptable scientific standard.

## References

[1] Chalcis sispes. Reference source
